# Reply

**DOI:** 10.1016/j.jvssci.2026.100453

**Published:** 2026-09-28

**Authors:** Salvatore T. Scali

**Affiliations:** Division of Vascular Surgery and Endovascular Therapy, University of Florida College of Medicine, Gainesville, FL

I am grateful to Liljeqvist et al^1^ for their thoughtful response and welcome the opportunity to continue this dialogue. Their reply clarifies several points raised in my invited commentary and further strengthens the interpretation of their findings. I want to reiterate my admiration for this work and for this group’s sustained contributions to aneurysm geometry, biomechanics, radiology, and molecular biology.

The authors appropriately emphasize that their conclusions extend beyond the relatively small number of intraluminal thrombus (ILT)-free aneurysms at surgery or rupture. The earlier baseline computed tomography angiography analysis of electively repaired aneurysms (n = 128, 27% with minimal or absent ILT) and the surveillance cohort of small aneurysms (n = 161, 15% without ILT) substantially enlarge the informative sample. Moreover, ILT thickness analyzed continuously was not significantly associated with growth, rupture, or progression to surgical threshold. The recovery of established associations, diabetes with slower growth and smoking with rupture, also supports the sensitivity of the dataset. Taken together, I agree that these analyses argue against a large, clinically meaningful association between ILT absence and aneurysm behavior.

Importantly, however, these findings do not exclude clinically relevant biological effects of thrombus itself. Maximal ILT thickness at a single cross-sectional level is a pragmatic radiographic measure, but it cannot capture total thrombus burden, spatial distribution, organization, permeability, hemorrhage, or regional relationships among thrombus, wall stress, and wall degeneration. As the authors note, ILT may exert countervailing effects, reducing mural wall stress while simultaneously promoting hypoxia, inflammation, proteolysis, and wall weakening. A present vs absent or thick vs thin classification may therefore be insufficient to resolve these competing biological processes.

Therefore, we are in agreement regarding what these data do and do not justify. They do not support altering repair thresholds solely on the basis of ILT absence. Their broader contribution is conceptual: abdominal aortic aneurysm likely represents a spectrum of biologically distinct remodeling phenotypes, and personalized management may ultimately require integrating diameter with geometry, biomechanics, thrombus phenotype, and wall biology.

The next step should be prospective multicenter investigation incorporating serial three-dimensional ILT segmentation, patient-specific biomechanical modeling, molecular imaging, circulating biomarkers, and spatially matched tissue profiling. This group is exceptionally well positioned to advance that work.

I congratulate the authors again on a rigorous and stimulating study and thank them for a collegial and constructive exchange.

## Funding

None.

## Disclosures

None.
